# CRT-D Implantation Through a Persistent Left Superior Vena Cava

**DOI:** 10.1016/s0972-6292(16)30759-8

**Published:** 2014-05-25

**Authors:** Rui Placido, Joao Sousa, Pedro Marques

**Affiliations:** Hospital Santa Maria, Serv Cardiologia I, Lisbon Academic Medical Centre, CCUL, Lisbon, Portugal

**Keywords:** CRT-D Implantation, Persistent Left Superior Vena Cava

A persistent left superior vena cava (PLSVC) was present in a 74 year-old man with dilated cardiomyopathy undergoing implantation of a cardiac resynchronization therapy device with defibrillator (CRT-D). A dual-coil active-fixation defibrillator lead was positioned in the right ventricular apex, followed by a SonR active fixation lead in the right atrial free wall. The coronary sinus lead was advanced into a postero-lateral vein (Figure 1, Panel A). All three leads were implanted through the PLSVC. The acute thresholds were normal. A chest X-ray was performed to confirm the lead positions (Figure 1, Panel B).

Persistent left superior vena cava (PLSVC) is the most common variation in the anomalous venous return to the heart, accounting for 0.2-4.3% of all congenital cardiac anomalies [1]. This anomaly is usually asymptomatic and unrecognized until left cephalic or subclavian approach is used for diagnostic [2,3]. It can pose particular difficulty when introducing electrodes in the heart chambers.

## Figures and Tables

**Figure 1:**
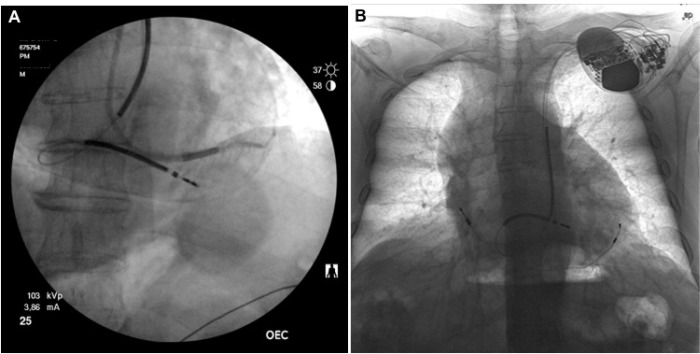
CRT-D Implantation Through a Persistent Left Superior Vena Cava
